# A Longitudinal Examination of Alcohol-Related Blackouts as a Predictor of Changes in Learning, Memory, and Executive Function in Adolescents

**DOI:** 10.3389/fpsyt.2022.866051

**Published:** 2022-05-06

**Authors:** Sara A. Lorkiewicz, Fiona C. Baker, Eva M. Müller-Oehring, Amie Haas, Robert Wickham, Stephanie A. Sassoon, Duncan B. Clark, Kate B. Nooner, Susan F. Tapert, Sandra A. Brown, Tilman Schulte

**Affiliations:** ^1^Clinical Psychology, Palo Alto University, Palo Alto, CA, United States; ^2^SRI International, Neuroscience Program, Menlo Park, CA, United States; ^3^Brain Function Research Group, School of Physiology, University of Witwatersrand, Johannesburg, South Africa; ^4^Department of Psychiatry and Behavioral Sciences, Stanford University School of Medicine, Stanford, CA, United States; ^5^Department of Neurology and Neurological Sciences, Stanford University School of Medicine, Stanford, CA, United States; ^6^Department of Psychological Sciences, Northern Arizona University, Flagstaff, AZ, United States; ^7^Department of Psychiatry, University of Pittsburgh, Pittsburgh, PA, United States; ^8^Department of Psychology, University of North Carolina, Wilmington, Wilmington, NC, United States; ^9^Department of Psychiatry, University of California, San Diego, La Jolla, CA, United States; ^10^Department of Psychology, University of California, San Diego, La Jolla, CA, United States

**Keywords:** blackouts, cognition, adolescents, memory, executive function, longitudinal, alcohol, development

## Abstract

**Introduction:**

In adolescents, the relationship between alcohol-related blackouts (ARBs) and distinct cognitive changes lasting beyond intoxication is unclear. We examined ARBs as a predictor of persistent changes in the development of learning, memory, and executive function in participants from the National Consortium on Alcohol and Neurodevelopment in Adolescence (NCANDA) study.

**Methods:**

Descriptive analyses of the NCANDA sample (*N* = 831, 50.9% female, 12–21 years at baseline) identified ARB patterns within participants with an ARB history (*n* = 106). Latent growth curve modeling evaluated ARB-related performance changes on four neuropsychological measures across five years, excluding baseline data to reduce the magnitude of practice effects over time (*n* = 790). Measures included the Penn Conditional Exclusion Test (PCET), Penn Letter N-back Test (PLBT), Penn Facial Memory Test immediate (PFMT_i_), and delayed (PFMT_d_) recognition trials, and the Rey Complex Figure Test copy (RCFT_c_), immediate recall (RCFT_i_), and delayed recall (RCFT_d_) trials. Multivariate models were fit for raw accuracy scores from each measure, with ARB history (i.e., presence of past-year ARBs) as the main independent variable. Age, sex, race, socioeconomic status, assessment site, and alcohol use (i.e., past-year frequency) were included as covariates. Interaction effects between ARB history and alcohol use frequency were tested.

**Results:**

By year five, 16% of participants had experienced at least one ARB (59% of whom reported > 1 ARB and 57% of whom had an ARB lasting > 1 h). After controlling for demographics and alcohol use, ARB history predicted attenuated PFMT_d_ performance growth at year one. Interaction effects between ARB history and alcohol use frequency predicted attenuated PFMT_d_ performance growth at years one and two. ARB history predicted attenuated RCFT_i_ and RCFT_d_ performance growth by year four, but not PCET or PLBT performance over time. By contrast, greater past-year alcohol use predicted attenuated PFMT_i_ and PFMT_d_ performance growth between years two and four in adolescents without an ARB history.

**Conclusion:**

We found that ARBs predict distinct, lasting changes in learning and memory for visual information, with results suggesting that the developing brain is vulnerable to ARBs during adolescence and emerging adulthood.

## Introduction

Alcohol-related blackouts (ARBs) are a type of anterograde amnesia for details of a drinking event during which an individual is consciously interacting with their environment (2). ARBs are particularly common among younger drinkers, with approximately 35% of high school students reporting at least one past-year ARB and 50% of college students reporting at least one lifetime ARB (3–6). Recent literature further suggests that ARBs are described as a neutral or positive experience, with some college students reporting drinking with the intention to blackout (7, 8). The perception that ARBs are a benign or even desired consequence of risky alcohol use is particularly noteworthy in the context of ARBs strongly predicting adverse, alcohol-related functional outcomes (e.g., social, emotional, occupational, and legal) in adolescents above and beyond alcohol use disorders (AUDs) *per se* (4, 9–11). Thus, consistently high ARB prevalence rates during key periods of adolescent/emerging adulthood development and their robust association with negative alcohol-related consequences have highlighted this phenomenon as a critical area of research.

Binge drinking [i.e., ≥5 drinks for males and ≥4 drinks for females over a 2-h period; (12)] and related behaviors common in younger drinking cohorts (e.g., pregaming and drinking games) strongly predict ARBs (13–15). Ultimately, ARBs occur due to a rapid rise in blood alcohol concentration (BAC) and subsequent disruption of long-term potentiation, the major neurochemical process considered to underlie memory formation or the transfer of sensory information from short-term memory to long-term storage (2). While ARBs are a marker of acute, alcohol-induced cognitive dysfunction, neuroimaging studies also describe blacking out as a possible indication of neurological vulnerability to heavy alcohol use and alcohol-related neurotoxicity in adolescents (16, 17). Findings suggest that ARBs are most commonly associated with persistent neurochemical and structural abnormalities in brain regions underlying executive function and learning and memory (18–20). However, functional imaging studies largely report ARBs as predictive of underlying neurological changes in the absence of overtly impaired performance on neuropsychological measures (21, 22). As such, behavioral data associated with ARB-related neurocognitive changes in adolescents are scarce.

The few studies examining relationships between ARBs and performance on cognitive measures in adolescents have focused on college students and rely on cross-sectional designs, which precludes a concrete determination of directionality. Findings are also inconsistent regarding patterns of ARB-related change in specific cognitive domains. For example, a study by Zamroziewicz et al. (23) found that, among college students, higher ARB frequency was associated with lower performance on an event-based prospective memory task. Findings from another study in college students by Min et al. (24) suggested that earlier ARB onset was related to lower non-contextual verbal memory abilities. In contrast, a longer duration of ARB history was associated with poor attention and executive function. Notably, neither study controlled for alcohol use, and the extent to which heavy, intermittent drinking confounded these results remains unclear. There is, therefore, a paucity of prior research exploring the relationship between ARBs and changes in cognition persisting beyond the period of acute alcohol intoxication which consider effects across the range of adolescence encompassing teenage years and early twenties. Further, to better identify the unique effects of ARBs on cognitive function in adolescents, longitudinal research that controls for alcohol use is required.

In the current study, we examined longitudinal data from the National Consortium on Alcohol and Neurodevelopment in Adolescence (NCANDA) study. The first aim was to identify ARB prevalence patterns from adolescence to emerging adulthood using cross-sectional data over six years of collection. The second aim was to expand the extant literature on ARB-associated cognitive change by using longitudinal data to identify how ARBs impact the development of cognition in adolescents over time while controlling for alcohol use frequency. Latent growth curve modeling (LGM), a reliable tool for studying cognitive development in cohorts across the lifespan, was used to explore whether stable, past-year ARB history during adolescents predicted attenuated cognitive development in domains of cognition shown to be sensitive to heavy, intermittent alcohol use during adolescents (25–30, 92). Specifically, we hypothesized that adolescents with a history of ARBs would demonstrate attenuated growth in performance on neuropsychological measures of (a.) executive functioning (i.e., mental flexibility and visual working memory) and (b.) learning and memory (i.e., facial episodic memory and visual learning and memory) across five years of data collection.

## Materials and Methods

### Study Sample

Participants were 831 adolescents and emerging adults (i.e., ages 12–21 years at study entry) recruited as part of the NCANDA study across five sites in the United States [for a full description of methods and baseline data, see Brown et al. (31)]. The study utilized an accelerated longitudinal design to capture developmental change across more age groups and data were collected to reflect the demographic characteristics of each site’s geographical location. Recruitment oversampled participants at risk for alcohol use initiation [i.e., drinking before age 15, family history of substance use disorder [SUD], ≥1 externalizing and ≥2 internalizing symptoms (32, 33)] and participants between ages 12 and 15 to better evaluate critical periods of growth (34, 35). Baseline inclusion criteria were age between 12 and 21 years, English fluency, and residence of <50 miles from assessment site.

Exclusion criteria were MRI contraindications, vision and/or hearing impairment, lack of parental consent, medications impacting brain function and/or blood flow, select medical conditions (i.e., those impacting MRI results, brain development, or study participation), early developmental problems, psychiatric disorders interfering with protocol completion (including substance use disorders), parental history of psychotic disorder independent of substance use, and developmental or severe learning disorder [see Brown et al. (31) for full description of inclusion and exclusion criteria]. While the majority of the sample had limited alcohol and other substance use exposure at baseline (NON-drinkers), 17% of participants were allowed to exceed baseline age-adjusted drinking, nicotine, and marijuana thresholds [exceeds threshold [ET]-drinkers (36, 37)] to ensure that individuals with a range of alcohol use severities were sampled to create comparison groups for later analyses. Compared to NON-drinkers (*n* = 692), ET-drinkers (*n* = 139) tended to be older (60% aged 18–21 years) and reported earlier age of alcohol use onset [25% reported drinking before age 15 (31)]. In the current study, both NON-and ET participants were included in the analysis.

Since the current analyses focused on effects of ARBs on neurocognitive development over time, it was critical to control for learning effects in the neuropsychological test battery. Findings from Sullivan et al. (1) suggest that significant learning effects exist in the NCANDA sample between baseline and the first annual follow-up which may confound future longitudinal analyses on developmental effects, a re-test pattern that has also been demonstrated in other studies (38) and in an analysis of executive function in the NCANDA sample (39). Baseline data were therefore excluded, resulting in an initial working sample of 790 participants.

### Measures and Procedures

Prior to study participation at each annual follow-up visit, all participants underwent informed consent procedures in which adult participants or parents of minor participants provided written informed consent and minor participants provided assent. The Institutional Review Boards of each site approved all NCANDA study protocols. At baseline and five annual follow-up visits, participants completed neuropsychological testing, neuroimaging sessions, and comprehensive psychosocial and psychodiagnostic assessments. Demographic data were collected at baseline and each annual follow-up [see Brown et al. (31) for full description of demographic measures].

In the current study, demographic factors known to impact performance on neuropsychological tests within the NCANDA sample were analyzed longitudinally as covariates, including age, sex, race, socioeconomic status (SES), and assessment site (1, 40–43). In longitudinal models, age was defined as the basis for individually varying times of observation for the outcomes and centered on 12 years (i.e., youngest age at study entry). In doing so, participant-specific variation in duration between annual assessments was controlled for. Age was therefore analyzed as a continuous metric impacting growth rather than as a categorical, fixed factor loading or traditional covariate, which is an important consideration for examining cognitive change during periods of rapid development (44–46). Baseline data for sex (i.e., dichotomous, male vs. female), race (i.e., dichotomous, white vs. non-white), and assessment site were included as time-invariant covariates (TICs). Baseline SES was included as a continuous TIC, defined as the highest level of parental education for either parent, an estimate known to be less sensitive to changes in family income due to geographical location, and was centered on median level of parental education (31, 40).

Past-year ARB and alcohol use history were assessed at baseline and each annual follow-up using the Customary Drinking and Drug Use Record (CDDR), an interviewer-administered, self-report questionnaire measuring current and past alcohol and other substance use [e.g., level of involvement, symptoms of withdrawal and dependence, and negative consequences (31, 47)]. The current study defined ARB history as the presence of ARBs in the past year (i.e., yes versus no) measured by a single CDDR item [i.e., “In the past year, have there been periods of time that later you could not remember while drinking alcohol/under the influence?” (5, 22)]. ARB history was included as a time-varying covariate (TVC) and main predictor of cognitive function in all longitudinal models. Past-year ARB frequency (i.e., number of ARBs in the past year at each annual follow-up) and duration of longest past-year ARB at each annual follow-up (i.e., ≥1 or <1 h) were analyzed cross-sectionally. To better distinguish between the effects of ARBs and alcohol use on cognition, alcohol use was controlled for and included in longitudinal models as a TVC. Alcohol use was defined as past-year alcohol use frequency (i.e., number of alcohol use days in the past year at each annual follow-up), a consumption item which reliably predicts alcohol use consequences in adolescents (48, 49). Past-year alcohol use was measured using a single CDDR item (i.e., “During the past year, how many days did you drink alcohol?”) and centered on mean number of days alcohol was used in the past year at each time point.

The NCANDA study’s full neuropsychological battery consisted of computerized and traditional measures administered at baseline and each annual follow-up selected based on reliability, minimization of practice effects, and adequate validity for age [see Sullivan et al. (1) for a detailed description of test selection]. All computerized measures are subtests of the Web-Based Computerized Neurocognitive Battery (WebCNP) which calculates raw accuracy and speed scores for each subtest and individual subtest trials (41). The current study analyzed data from three WebCNP subtests and one traditional test measuring components of the broader NCANDA target domains of executive function and learning and memory (see Table 1 for a summary of neuropsychological measures used). Raw accuracy scores were modeled as the main continuous, dependent variables in longitudinal analyses. Given that demographic covariates were controlled for in each model, raw scores were considered an accurate representation of each cognitive outcome in the absence of demographic or standardized corrections.

**TABLE 1:** Neuropsychological measures and associated cognitive domains.

NCANDA target domain	Neuropsychological measure	Cognitive abilities
Executive function	Penn Conditional Exclusion Test (PCET)^a^	Mental flexibility
	Penn Letter N-back Test (PLBT)^a^	Visual working memory
Learning and memory	Penn Facial Memory Test (PFMT)^a^	Facial episodic memory
	Immediate trial (PFMT_i_)	Immediate facial recognition
	Delayed trial (PFMT_d_)	Delayed facial recognition
	Rey Complex Figure Test (RCFT)^b^	Visual incidental learning and memory
	Copy trial (RCFT_c_)	Psychomotor function, visuospatial function,
		executive function (e.g., organization, planning)
	Immediate recall trial (RCFT_i_)	Incidental learning
	Delayed recall trial (RCFT_d_)	Delayed visual recall

*^a^Subtest of the WebCNP. ^b^Traditional paper and pencil measure.*

#### Measures of Executive Function

Raw accuracy scores from the Penn Conditional Exclusion Test (PCET) were modeled to measure ARB-related changes in mental flexibility, or set-shifting (41, 50). For the PCET, participants must use one of three sorting principles (i.e., size, shape, and line thickness) to determine which object among four others does not belong using only feedback given after each trial (i.e., “correct” or “incorrect”) and little other instruction. After ten successful trials, the sorting rule is changed, and participants must use hypothesis testing to determine the next rule (41). PCET accuracy scores were calculated by dividing total learning (i.e., product of correct responses and number of learned rules) by the product of total correct responses and total errors (51). Raw Penn Letter N-Back Test (PLBT) accuracy scores were modeled to measure ARB-related changes in visual working memory. On the PLBT, participants are presented with a series of letters one at a time, and in three separate conditions, asked to press a spacebar when (a.) target stimuli are shown (i.e., “0-back”), (b.) target letters match the previous letter (i.e., “1-back”), and (c.) target letters match the letter presented two letters back [i.e., “2-back” (41, 52)]. PLBT accuracy scores were defined as total number of correct responses.

#### Measures of Learning and Memory

Raw Penn Facial Memory Test (PFMT) accuracy scores were modeled to measure ARB-related changes in facial episodic memory. On the PFMT, participants are shown 20 digitized faces one at a time and are asked to identify them among 20 distractor faces immediately and again 20 min later for immediate (PFMT_i_) and delayed (PFMT_d_) recognition trials, respectively (41, 51, 53). For all recognition trials, participants are asked whether they had seen each face before using a four-choice scale [i.e., “definitely not,” “probably not,” “probably yes,” and “definitely yes;” (41)]. PFMT_i_ and PFMT_d_ accuracy scores were defined as total true positive responses and included as dependent variables in separate models (41). Raw accuracy scores from all three trials of the Rey Complex Figure Test (RCFT) were modeled to measure ARB-related changes in visual incidental learning and memory. The initial RCFT copy trial (RCFT_c_) measures more basic cognitive processes such as psychomotor, visuospatial, and executive functions (54, 55). For RCFT immediate (RCFT_i_) and delayed (RCFT_d_) recall trials, participants draw the figure from memory immediately after the copy trial and approximately 30 min after the immediate recall trial, respectively. Accuracy scores for all three RCFT trials were calculated based on total number of correctly drawn figure details and included as dependent variables in separate models.

### Data Analyses

SPSS (Version 27) was used to calculate cross-sectional, descriptive statistics for demographics and past-year alcohol use in participants with an ARB history at each annual visit between baseline and fifth-year follow-up. Bivariate comparisons were also made using SPSS to examine differences at each annual follow-up in demographics and alcohol use history between participants with (ARB^+^) and without (ARB^–^) an ARB history using two-tailed, independent samples *t*-tests for continuous variables (i.e., age, SES, and alcohol use) and chi-square tests of independence for categorical variables (e.g., sex, race, and assessment site). Past-year ARB frequency and duration variables were analyzed cross-sectionally at each annual visit (i.e., baseline through fifth-year follow-up) to better describe ARB patterns within the NCANDA sample. ARB frequency and duration were excluded from longitudinal models due to low base rates of participants reporting >2 ARBs (4.8% of total sample at the fifth annual follow-up) and collinearity between ARB history (yes/no) and longest duration. As such, only past-year ARB history (yes/no) was analyzed longitudinally as the main independent variable. Longitudinal analyses were conducted using the *MPlus* version 8.5 with the robust Full Information Maximum Likelihood (FIML) algorithm for missing data (56). A series of LGM were specified to model the development of cognition over time and predict how individual and group demographic factors and alcohol use characteristics impacted participant performance on select neuropsychological measures across 5 years of data collection (57). The LGM intercept and slope parameters describe the relationship between ARBs and cognition over time and evaluate the hypotheses that ARB history is associated with attenuated development of executive function and learning and memory between the first and fifth annual follow-up assessments.

### Missing Data

**FIGURE 1:**
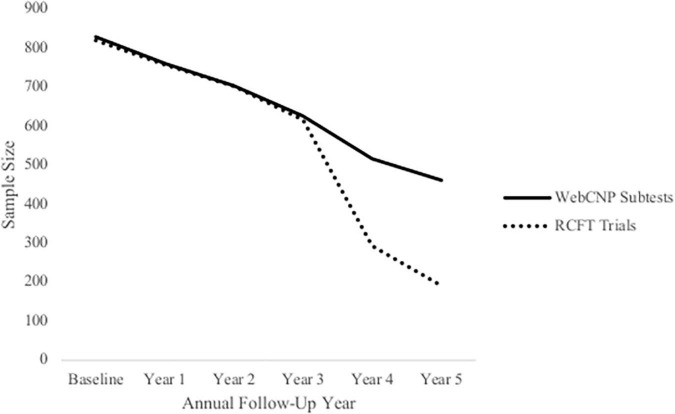
Attrition within the National Consortium on Alcohol and Neurodevelopment in Adolescence (NCANDA) sample. This figure depicts sample size at each annual follow-up across 5 years based on participants who completed WebCNP measures (solid line) and all RCFT trials (dotted line) measures.

Figure 1 depicts the progression of attrition over time based on the number of participants who completed WebCNP measures and RCFT trials at each annual follow-up. Analyses of missing data between baseline and the fifth annual follow-up found a 5-year, six-occasion retention rate of approximately 56% (i.e., subjects without any missing data). Sample size at each annual follow-up for WebCNP measures were similar, with greater variation observed between WebCNP measures and RCFT trials at later assessment timepoints. No significant relationships were found between dropout and past-year alcohol use or ARB history. The FIML estimator in *MPlus* uses all available data without excluding cases by list wise deletion and provides unbiased estimates of model parameters under the conditional missing at random assumption (58–60). Since FIML only estimates missing data for dependent variables, multiple imputation (number of imputations = 10) accounted for missing data on all independent variables (61).

### Growth Curve Fit

Both linear and quadratic models were considered during model selection. Separate growth curves were fit for raw accuracy scores from each neuropsychological measure as well as individual trials yielding unique accuracy scores. Unconditional models (i.e., models which do not include independent variables or covariates) were compared using fit statistics from the Akaike Information Criterion [AIC; Akaike (62)], Bayesian Information Criterion [BIC; Nylund et al. (63)], Root Mean Squared Error of Approximation [RMSEA; Hancock and Freeman (64)], and standard root mean square residual [SRMR; Hancock and Mueller (57)]. Model selection was based on a combination of the lowest AIC and BIC values, RMSEA and SRMR values < 0.08, and interpretability. Table 2 depicts fit statistics for final unconditional models organized by cognitive domain and neuropsychological measure. Final unconditional models for accuracy scores from measures of executive function (i.e., the PCET and PLBT) were modeled using linear functions while scores from measures of learning and memory (i.e., the PFMT and RCFT) followed a quadratic functional form.

**TABLE 2:** Fit statistics for best-fit unconditional models.

	Goodness of fit statistics							
	
	Linear				Quadratic			
		
Unconditional models	AIC^a^	BIC^a^	RMSEA^b^	SRMR^c^	AIC	BIC	RMSEA	SRMR
Penn Conditional Exclusion Test^†^	**–7309.3**	**–7243.9**	**0.000**	**0.017**				
Penn Letter N-back Test	**13628.6**	**13675.3**	**0.015**	**0.056**	13634.4	13699.8	0.029	0.052
Penn Facial Memory Test								
Immediate trial	14382.2	14428.9	0.072	0.060	**14335.6**	**14401.0**	**0.000**	**0.016**
Delayed trial	14328.3	14375.0	0.034	0.092	**14313.2**	**14378.6**	**0.000**	**0.077**
Rey Complex Figure Test								
Copy Trial	13338.3	13385.0	0.035	0.172	**13333.9**	**13399.2**	**0.028**	**0.073**
Immediate Recall Trial	14855.8	14902.5	0.034	0.067	**14854.3**	**14919.7**	**0.028**	**0.043**
Delayed Recall Trial	14771.2	14817.8	0.041	0.078	**14768.8**	**14834.1**	**0.036**	**0.032**

*Boldface type indicates best fit models. ^a^Lower values suggest better fit. ^b^RMSEA and SRMR of ≤0.08 suggest good model fit. ^†^PCET data did not converge when fitting quadratic models.*

**FIGURE 2:**
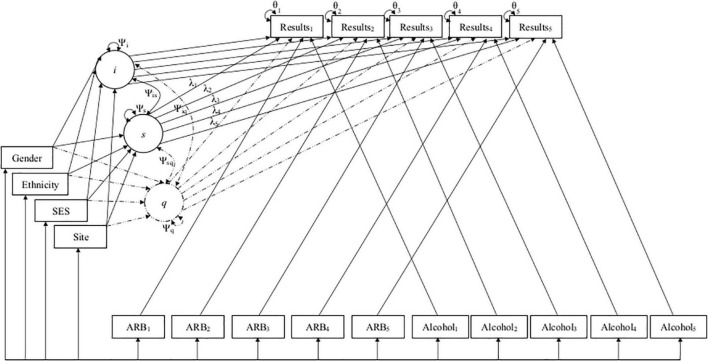
Path diagrams for linear and quadratic conditional models. This figure depicts the path diagrams for linear and quadratic growth models, with solid lines depicting the original linear model and dashed lines representing additional parameters added to create the final quadratic model. Linear and quadratic models estimated the latent intercept (*i*), linear slope (*s*), and quadratic slope (*q*) growth factors to predict how individual and group characteristics (i.e., demographics and past-year alcohol use frequency and ARB history) impacted development of cognition across five timepoints. Growth factors *i*, *s*, and *q* are depicted in circles along with their variances (Ψ*_*i*_*,Ψ*_*s*_*,Ψ*_*q*_*) and co-variances (Ψ*_*is*_*,Ψ*_*iq*_*,Ψ*_*sq*_*). Demographics are included as time-invariant covariates (TICs) in boxes on the left of the figure. Time was parameterized using age as individually varying times of observation (λ_1–5_). ARB history (ARB_1–5_) and alcohol use frequency (Alcohol_1–5_) are included as time-varying covariates (TVCs) in boxes at the bottom of the figure. Observed neuropsychological test scores (Results_1–5_) and their residual variance (θ_1–5_) at each time point are included in boxes at the top of the figure.

Using unconditional growth models, intercept means and variances estimated average initial test performance at the first annual follow-up and variation between participants in first year task performance. Linear slope means and variances estimated average annual growth in test performance and variability of linear growth rate between participants, respectively. Quadratic slope means estimated the magnitude to which growth in neuropsychological test performance tapered off or accelerated over time, with variances estimating overall diversity in quadratic growth rate. Covariances between intercept and slopes estimated the degree to which the rate of linear or quadratic growth related to initial test performance as well as how linear and quadratic slopes impacted the growth rates of one another. Once unconditional models were identified, the relationship between both TICs and TVCs, initial neuropsychological task performance, and subsequent performance growth over time was then evaluated in conditional models. All covariates were examined independently and in a multivariate manner by including them into each model in a stepwise fashion. Age was included first to control for individually varying times of observation, followed by all TICs in the order of (1.) gender, (2.) ethnicity, (3.) SES, and (4.) assessment site. TVCs were subsequently included in each model a similar step-wise manner (i.e., past-year alcohol use frequency was added first followed by past-year ARB history [yes/no]) to examine their impact on neuropsychological task performance over time while controlling for TICs. Finally, the interaction between ARB history and alcohol use frequency was assessed in final multivariate, conditional models. Path diagrams for linear and quadratic models are displayed in Figure 2. Path diagrams for models with an interaction are omitted due to complexity.

## Results

### Characteristics of National Consortium on Alcohol and Neurodevelopment in Adolescence ARB^+^ Participants

**FIGURE 3:**
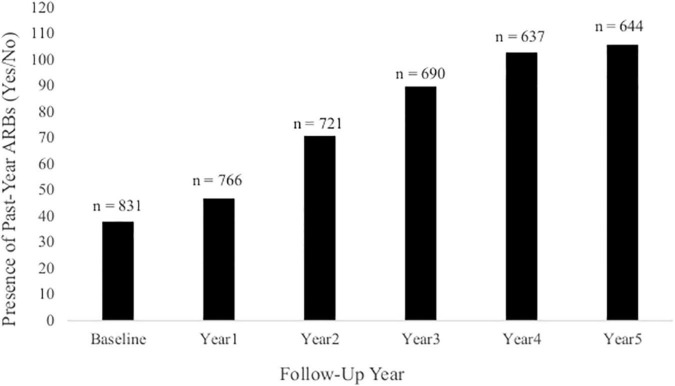
The presence of past-year ARBs (yes/no) at each annual follow-up. This figure illustrates the frequency of participants at each annual follow-up who had experienced at least one past-year ARB. Total sample size at each annual follow-up is indicated above each column.

**FIGURE 4:**
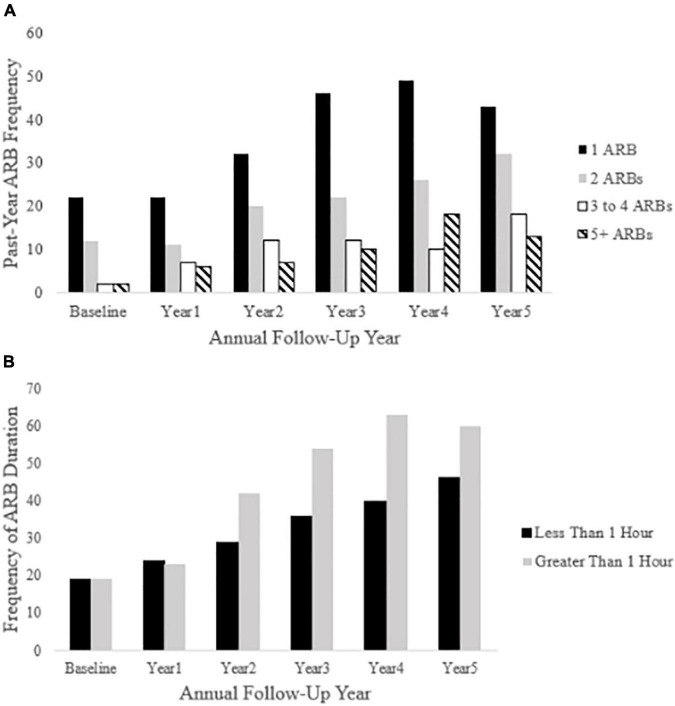
Past-year ARB frequency and longest duration at each annual follow-up. Panel **(A)** depicts ARB frequency at each annual follow-up (baseline through year five). Panel **(B)** depicts ARB duration (less than 1 h or greater than 1 h) in participants reporting a past-year ARB history.

**FIGURE 5:**
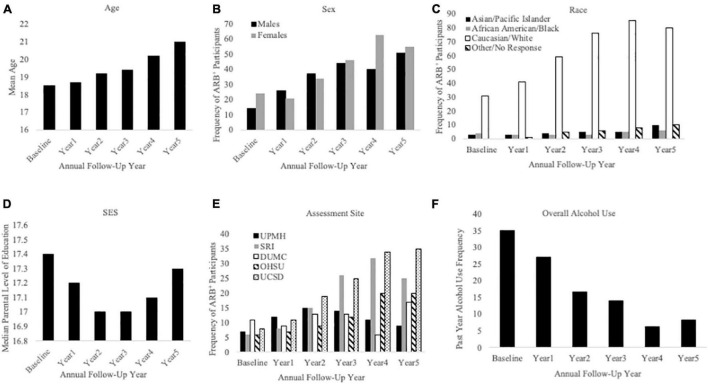
Sample characteristics for participants reporting a past-year ARB history (ARB^+^ participants). This figure describes ARB^+^ participant sample characteristics for demographics and alcohol use frequency at baseline and each annual follow-up. The x-axis is defined as annual follow-up year in panels **(A)** through **(F)**. Panel **(A)** shows mean age of ARB^+^ participants, panel **(B)** shows ARB^+^ participants by sex, and panel **(C)** shows ARB^+^ participants by race. Panel **(D)** depicts ARB^+^ participants by median level of parental education, panel **(E)** depicts ARB^+^ participants by assessment site, and panel **(F)** depicts past-year alcohol use frequency.

Cross-sectional, frequency data for having experienced at least one past-year ARB (yes/no) at baseline and each annual follow-up are depicted in Figure 3. Cross-sectional frequency data for past-year ARB frequency and longest duration at baseline and each annual follow-up are summarized in Figure 4. At baseline, 38 participants reported at least one ARB in the past-year (4.6% of total sample). Baseline ARB^+^ individuals most often reported one past-year ARB (57.9%) and 50% described their longest ARB as ≥1 h. By the fifth-year annual follow-up, 16.4% of the total sample (*n* = 106) endorsed at least one past-year ARB, with less than half of participants endorsing only a single past-year ARB (43%) and 56.6% describing their longest ARB as > 1 h. ARB^+^ participant characteristics for age, sex, race, SES, assessment site, and alcohol use frequency are presented in Figure 5. At baseline, mean age of ARB^+^ participants was 18.5 (SD = 1.8) years, 63.2% of participants were male, 81.6% were White, mean SES was 17.4 (SD = 2.2) years of parental education, and average number of past-year drinking days was 35.1 (SD = 41.9). By the fifth annual follow-up, mean age for ARB^+^ participants was 21.0 (SD = 2.0) years, 51.9% of participants were male, 75.5% were White, mean SES was 17.3 (SD = 2.3) years of parental education, and average number of past-year drinking days was 80.07 (SD = 57.24). Results from bivariate comparisons between ARB^+^ and ARB^–^ participant characteristics are presented in Table 3. ARB history was associated with higher alcohol use frequency at all time points (*p* = 0.01) and, compared to ARB^–^ participants, ARB^+^ participants were significantly older (*p* = 0.01) between baseline and the fourth annual follow-up. Inconsistent, significant differences were also found between ARB^+^ and ARB^–^ participants in sex, race, and assessment site. No significant differences in SES were found between ARB^+^and ARB^–^ participants at any time point.

**TABLE 3:** Bivariate comparisons between ARB^+^ and ARB^–^ participant characteristics at baseline and each annual follow-up.

	Baseline	Year 1	Year 2	Year 3	Year 4	Year 5
Age^b^	9.55 (819)**	7.20 (764)**	7.69 (719)**	3.44 (688)**	2.72 (635)**	1.66 (642)
Sex^a^	2.34 (1)	0.39 (1)	0.06 (1)	0.16 (1)	6.04 (1)**	0.50 (1)
Race^a^	18.56 (6)**	7.79 (6)	13.00 (6)*	15.32 (6)*	17.54 (6)**	6.88 (6)
SES*^b^*	1.53 (818)	1.17 (764)	0.69 (710)	0.69 (688)	1.51 (635)	1.86 (641)
Assessment site^a^	2.36 (4)	4.44 (4)	4.38 (4)	9.66 (4)*	25.88 (4)**	10.04 (4)*
Overall alcohol use^b^	4.71 (37.27)**	6.40 (46.85)**	8.85 (74.57)**	8.42 (100.58)**	8.13 (100.37)**	8.14 (138.93)**

^a^Chi-square test (χ^2^) reported as χ^2^(df). ^b^Independent samples t-test reported as t(df); **p < 0.01; *p < 0.05.

### ARB-Related Changes in Executive Function and Learning and Memory

#### Unconditional Growth Model Parameter Estimates: Executive Function

Unconditional model parameter estimates for performance on measures of mental flexibility (i.e., PCET accuracy scores) and visual working memory (i.e., PLBT accuracy scores) are presented in Table 4. Initial PCET performance was 0.278 (SE = 0.003), *p* = 0.00, points on average and was predicted to increase linearly by 0.008 (SE = 0.001), *p* = 0.00, points per year. Average initial PLBT performance was 28.4 (SE = 0.06), *p* = 0.01, points, with significant variability found between participants. Linear PLBT performance growth was estimated to increase by 0.02 (SE = 0.27) points per year and was not significant (*p* = 0.39).

**TABLE 4:** Intercept and slope factor parameter estimates for unconditional models.

Model Part	Parameter	Parameter estimate (SE^†^)						
		
		PCET^a^	PLBT^b^	PFMT_i_^c^	PFMT_d_^d^	RCFT_c_^e^	RCFT_i_^f^	RCFT_d_^g^
**Growth factor means**								
Intercept	*i*	0.278 (0.003)**	28.446 (0.059)**	34.956 (0.116)**	34.731 (0.181)**	30.545 (0.132)**	21.274 (0.343)**	22.252 (0.212)**
Linear slope	*s*	0.008 (0.001)**	0.025 (0.029)	0.901 (0.087)**	0.829 (0.124)**	−0.312 (0.130)*	1.031 (0.251)**	0.655 (0.163)**
Quadratic slope	*q*			−0.154 (0.022)**	−0.098 (0.021)**	0.093 (0.036)*	−0.066 (0.045)	−0.010 (0.043)
**Growth factor variance**								
Intercept	ψ*_*i*_*	0.003 (0.001)	1.135 (0.273)**	6.928 (0.794)**	6.296 (2.613)*	7.609 (1.313)**	33.505 (8.226)**	26.693 (2.292)**
Linear slope	ψ*_*s*_*	0.000 (0.000)	0.079 (0.052)	0.212 (0.539)	−0.269 (1.163)	1.086 (1.226)	8.022 (3.532)*	4.628 (1.804)**
Quadratic slope	ψ*_*q*_*			0.013 (0.029)	−0.009 (0.031)	0.027 (0.061)	0.214 (0.094)*	0.206 (0.093)*
**Growth factor covariance**								
Intercept	ψ*_*is*_*	0.000 (0.000)	−0.054 (0.086)	−0.070 (0.579)	0.070 (1.676)	−0.287 (1.200)	−8.721 (5.223)	−2.690 (1.854)
Linear Slope	ψ*_*iq*_*			−0.016 (0.125)	−0.060 (0.252)	−0.114 (0.257)	1.190 (0.791)	0.216 (0.405)
Quadratic Slope	ψ*_*sq*_*			−0.030 (0.119)	0.068 (0.184)	−0.148 (0.263)	−1.264 (0.559)*	−0.899 (0.393)*

**^†^**Standard error. ^a^Penn Conditional Exclusion Test. ^b^Penn Letter N-back Test. ^c^Penn Facial Memory Test, immediate recognition trial. ^d^Penn Facial Memory Test, delayed recognition trial. ^e^Rey Complex Figure Test, copy trial. ^f^Rey Complex Figure Test, immediate recall trial. ^g^Rey Complex Figure Test, delayed recall trial. **p < 0.01. *p < 0.05.

#### Unconditional Growth Model Parameter Estimates: Learning and Memory

Unconditional model parameter estimates for performance on measures of facial episodic memory (i.e., PFMT trial) and visual incidental learning and delayed recall (i.e., RCFT trial) are presented in Table 4. Average initial PFMT_i_ and PFMT_d_ scores were 34.9 (SE = 0.12), *p* = 0.01, and 34.7 (SE = 0.18), *p* = 0.01, points, respectively, with significant variability in performance found between participants on both trials. PFMT_i_ and PFMT_d_ performance growth was predicted to increase linearly by 0.90 (SE = 0.54), *p* = 0.01, and 0.83 (SE = 0.12), *p* = 0.01, points linearly per year, respectively. For both the PFMT_i_ and PFMT_d_, quadratic performance growth was predicted to accelerate by 0.10 (*p* < 0.01), points per year at later annual follow-up visits. Average initial RCFT_c_ performance was 30.5 (SE = 0.13), *p* = 0.01, points, and predicted to decrease at rate of -0.31 (SE = 0.13), *p* = 0.02, points per year. Significant variability was found between participants for both RCFT_c_ intercept and slope parameter estimates. Quadratic RCFT_c_ performance growth predicted a 0.09 (SE = 0.04), *p* = 0.01, point accelerated annual increase at later follow-up visits. Average RCFT_i_ performance was 21.27 (SE = 0.34), *p* = 0.01 points and was predicted to increase linearly by 1.03 (SE = 0.25), *p* = 0.01, points per year. Both RCFT_i_ intercept and slope parameters varied significantly between participants and the covariance between RCFT_i_ linear and quadratic growth factors was significant, -1.26 (SE = 0.56), *p* = 0.02, suggesting that with faster linear growth, quadratic growth was attenuated. Average RCFT_d_ performance was 22.2 (SE = 0.21), *p* = 0.01, points and was predicted to increase linearly by 0.65 (SE = 0.16), *p* = 0.01, points per year. Both RCFT_d_ intercept and slope parameters varied significantly between participants and the covariance between RCFT_d_ linear and quadratic growth factors was significant, -0.90 (SE = 0.39), *p* = 0.02.

#### Conditional Growth Model Parameter Estimates: Executive Function

**FIGURE 6:**
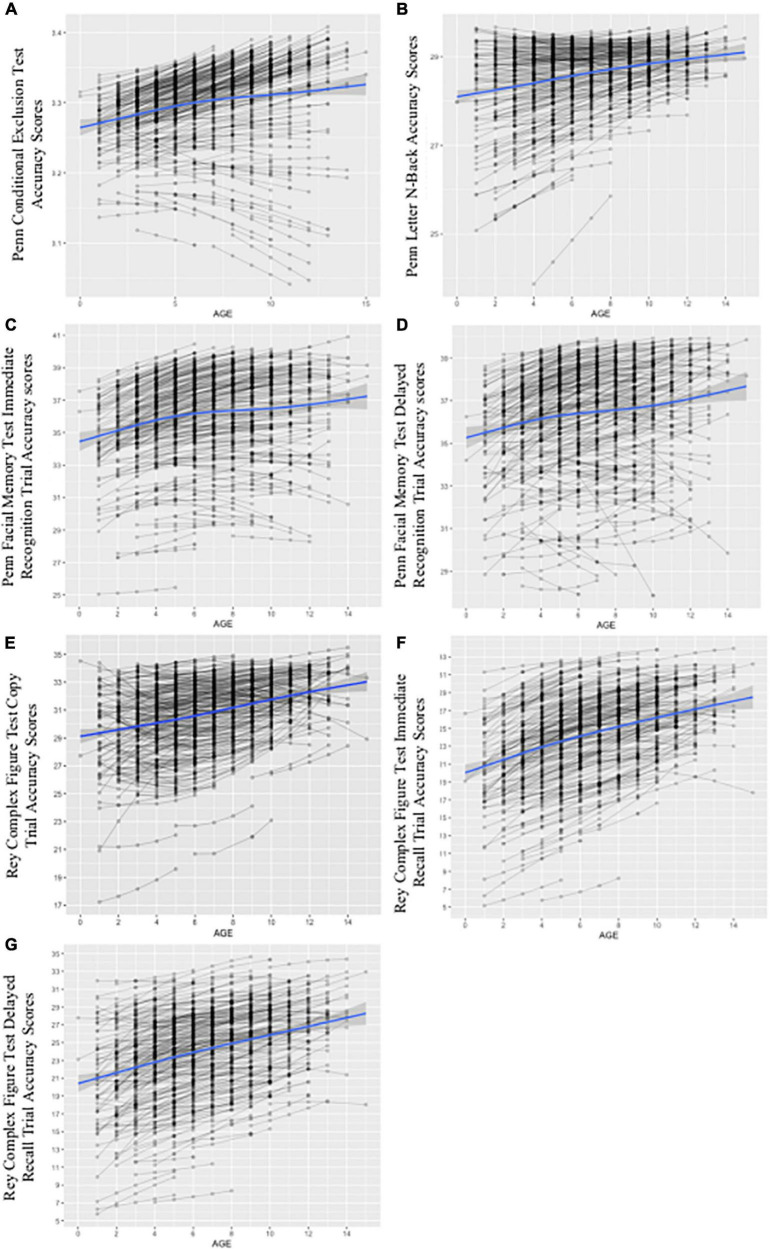
Growth curves for conditional models including time-invariant covariates (TICs) and time-varying covariates (TVCs). The figure depicts spaghetti plots for final, multivariate growth curve models including all TICs (i.e., demographics) and TVCs (i.e., past-year alcohol use frequency and ARB history) for the PCET in panel **(A)**, PLBT in panel **(B)**, PFMT_i_ in panel **(C)**, PFMT_d_ in panel **(D)**, RCFT_c_ in panel **(E)**, RCFT_i_ in panel **(F)**, and RCFT_d_ in panel **(G)**. The x-axis for panels **(A)** through **(G)** represents age as individually varying times of observation at each annual follow-up visit centered at 12 years, or youngest age at study entry. The y-axis represents respective test score scales with PCET scores ranging from 0 to 1 point, PLBT scores ranging from 0 to 30 points, PFMT scores ranging from 0 to 40 points, and RCFT trial scores ranging from 0 to 36 points. Individual participant test trajectories are represented by gray lines while the estimated growth curve is represented by the blue line with standard error shown in gray.

Figure 6 depicts final multivariate growth curve models including all TICs and TVCs for neuropsychological test performance for all measures between the first through fifth annual follow-ups. Intercept and slope factor parameter estimates for TICs from conditional growth models of performance on measures of mental flexibility (i.e., the PCET) and visual working memory (i.e., the PLBT) including only TICs are summarized in Table 5. SES (i.e., higher median parental education) predicted accelerated linear growth in PCET performance at a rate of 0.001 [SE = 0.000], *p* = 0.026, points. Sex and race were not significantly associated with PCET performance. Being White predicated accelerated growth in linear PLBT performance at a rate of 0.107 [SE = 0.045], *p* = 0.018, points per year. Sex, SES, and assessment site were not significantly associated with PLBT performance. Slope factor parameter estimates for TVCs from conditional growth models of performance on measures of set-shifting and visual working memory including both TICs and TVCs are summarized in Table 6. Initial performance and growth in performance on the PCET and PLBT were not significantly associated with alcohol use frequency, ARB history, or the interaction between alcohol use frequency and ARB history.

**TABLE 5:** Intercept and slope factor parameter estimates for time invariant covariates (TICs).

Neuropsychological test	Parameter	Gender	Ethnicity	SES
PCET^a^	*i*	0.009 (0.008)**^†^**	0.012 (0.010)	0.003 (0.002)
	*s*	−0.001 (0.001)	0.002 (0.002)	0.001 (0.000)*
PLBT^b^	*i*	−0.016 (0.220)	−0.076 (0.259)	0.049 (0.047)
	*s*	−0.007 (0.032)	0.107 (0.045)*	0.005 (0.007)
PFMT_i_^c^	*i*	0.276 (0.499)	−0.317 (0.532)	0.251 (0.111)*
	*s*	−0.119 (0.152)	0.272 (0.173)	−0.027 (0.035)
	*q*	0.002 (0.012)	−0.014 (0.013)	0.002 (0.003)
PFMT_d_^d^	*i*	−0.068 (0.522)	0.387 (0.518)	0.264 (0.106)*
	*s*	−0.173 (0.164)	−0.022 (0.164)	−0.055 (0.032)
	*q*	0.011 (0.013)	0.006 (0.013)	0.005 (0.002)*
RCFT_c_^e^	*i*	−0.123 (0.724)**^†^**	0.503 (0.798)	0.399 (0.151)**
	*s*	−0.052 (0.227)	−0.094 (0.252)	−0.020 (0.046)
	*q*	0.001 (0.017)	0.010 (0.019)	−0.001 (0.003)
RCFT_i_^f^	*i*	0.101 (0.851)	0.081 (0.939)	0.773 (0.198)**
	*s*	0.084 (0.251)	0.178 (0.285)	−0.063 (0.053)
	*q*	−0.014 (0.019)	0.007 (0.023)	0.001 (0.004)
RCFT_d_^g^	*i*	−1.214 (0.947)	−0.388 (1.039)	0.693 (0.213)**
	*s*	0.313 (0.277)	0.298 (0.302)	−0.045 (0.057)
	*q*	−0.021 (0.021)	0.001 (0.023)	0.001 (0.004)

Boldface font indicates significant findings. Conditional models from which results are displayed included only TICs. ^a^Penn Conditional Exclusion Test. ^b^Penn Letter N-back Test. ^c^Penn Facial Memory Test, immediate recognition trial. ^d^Penn Facial Memory Test, delayed recognition trial. ^e^Rey Complex Figure Test, copy trial. ^f^Rey Complex Figure Test, immediate recall trial. ^g^Rey Complex Figure Test, delayed recall trial. i, intercept factor. s, linear slope factor. q, quadratic slope factor. **^†^**Parameter Estimate (Standard Error). **p < 0.01. *p < 0.05.

**TABLE 6:** Slope factor parameter estimates for time-varying covariates (TVCs).

	Time-varying covariates	Annual follow-up visit				
		
		Year 1	Year 2	Year 3	Year 4	Year 5
PCET^a^	Alcohol use frequency	0.000 (0.000)**^†^**	0.000 (0.000)	0.000 (0.000)	0.000 (0.000)	0.000 (0.000)
	ARB history	−0.018 (0.012)	−0.011 (0.009)	−0.013 (0.011)	−0.001 (0.010)	0.005 (0.009)
	Interaction	0.000 (0.000)	0.000 (0.000)	0.000 (0.000)	0.000 (0.000)	0.000 (0.000)
PLBT^b^	Alcohol use frequency	0.004 (0.003)	−0.001 (0.003)	−0.002 (0.003)	0.002 (0.002)	0.002 (0.003)
	ARB history	−0.198 (0.245)	0.036 (0.209)	0.118 (0.239)	0.118 (0.210)	0.038 (0.203)
	Interaction	0.006 (0.007)	−0.002 (0.005)	0.001 (0.006)	0.002 (0.004)	−0.003 (0.005)
PFMT_i_^c^	Alcohol use frequency	−0.003 (0.006)	–0.010 (0.003)**	–0.008 (0.004)*	–0.007 (0.003)**	−0.003 (0.002)
	ARB history	−0.876 (0.454)	0.284 (0.290)	0.348 (0.256)	0.147 (0.259)	−0.229 (0.303)
	Interaction	−0.002 (0.013)	0.003 (0.007)	0.003 (0.006)	0.002 (0.005)	−0.008 (0.006)
PFMT_d_^d^	Alcohol use frequency	0.002 (0.006)	–0.012 (0.003)**	–0.007 (0.003)*	–0.007 (0.003)*	0.000 (0.003)
	ARB history	–1.489 (0.393)**	0.431 (0.285)	0.362 (0.226)	0.234 (0.273)	0.177 (0.246)
	Interaction	–0.03 (0.01)*	–0.02 (0.01)**	0.00 (0.00)	0.00 (0.01)	−0.01 (0.01)
RCFT_c_^e^	Alcohol use frequency	−0.002 (0.008)**^†^**	0.000 (0.005)	0.006 (0.004)	0.001 (0.005)	−0.003 (0.006)
	ARB history	0.954 (0.530)	0.186 (0.411)	−0.395 (0.365)	−0.123 (0.543)	1.569 (0.931)
	Interaction	−0.001 (0.016)	−0.005 (0.011)	−0.003 (0.008)	0.011 (0.010)	−0.005 (0.023)
RCFT_i_^f^	Alcohol use frequency	0.00 (0.01)	0.01 (0.01)	0.00 (0.00)	−0.01 (0.01)	−0.02 (0.01)
	ARB history	−0.26 (0.60)	−0.89 (0.61)	−0.12 (0.49)	–1.35 (0.68)*	1.62 (1.32)
	Interaction	0.02 (0.02)	0.02 (0.01)	0.00 (0.01)	0.00 (0.01)	0.01 (0.04)
RCFT_d_^g^	Alcohol use frequency	0.01 (0.01)	0.00 (0.01)	0.01 (0.00)	0.00 (0.00)	−0.02 (0.01)
	ARB history	−0.12 (0.61)	−0.43 (0.55)	−0.47 (0.47)	–1.07 (0.53)*	1.38 (1.18)
	Interaction	−0.01 (0.02)	0.01 (0.01)	0.00 (0.01)	0.01 (0.01)	−0.02 (0.04)

Boldface text indicates significant values. Conditional models from which results are displayed included both TICs and TVCs. ^a^Penn Conditional Exclusion Test. ^b^Penn Letter N-back Test. ^c^Penn Facial Memory Test, immediate recognition. ^d^Penn Facial Memory Test, delayed recognition. ^e^Rey Complex Figure Test, copy trial. ^f^Rey Complex Figure Test, immediate recall trial. ^g^Rey Complex Figure Test, delayed recall trial. **^†^**Parameter estimate (standard error). **p < 0.01. *p < 0.05.

#### Conditional Growth Model Parameter Estimates: Learning and Memory

Intercept and slope parameter estimates for TICs from conditional growth models of performance on measures of facial episodic memory (i.e., PFMT trials) and visual incidental learning and delayed recall (i.e., RCFT trials) including only TICs are summarized in Table 5. SES predicted higher initial PFMT_i_ and PFMT_d_ scores by 0.251 (SE = 0.111), *p* = 0.024, and 0.264 (SE = 0.106), *p* = 0.013, points, respectively. There were no significant associations between sex or race and the PFMT_i_ or PFMT_d_. SES predicted a higher initial RCFT performance on all three trials (*p* = 0.01). Sex and race did not significantly predict changes in initial performance or growth on any RCFT trials. Slope factor parameter estimates for TVCs from conditional growth models of performance on measures of facial episodic memory and visual incidental learning and delayed recall including both TICs and TVCs are summarized in Table 6. Higher alcohol use frequency predicted significantly attenuated growth in PFMT_i_ and PFMT_d_ performance between the second and fourth annual follow-up visits. ARB history and its interaction with alcohol use frequency were not significantly associated with changes in PFMT_i_ performance. However, compared to ARB^–^ participants, experiencing at least one past-year ARB predicted attenuated annual linear growth in PFMT_d_ performance by -1.50 (SE = 0.39), *p* = 0.01, points per year above and beyond alcohol use frequency by the first annual follow-up. The interaction between ARB history and alcohol use frequency significantly predicted attenuated linear growth in PFMT_d_ performance between the first and second annual follow-ups. Additionally, compared to ARB^–^ participants, experiencing at least one past-year ARB significantly predicted attenuated annual linear growth in both RCFT_i_ and RCFT_d_ performance of -1.35 (SE = 0.68), *p* = 0.04, and -1.07 (SE = 0.5), *p* = 0.04, points per year respectively, above and beyond alcohol use frequency by the fourth annual follow-up visit. ARB history was not significantly associated with RCFT_c_ performance. Alcohol use frequency and its interaction with ARB history were not significantly associated with performance on any RCFT trials.

## Discussion

Using the large, adolescent-focused NCANDA study, we found that one of every six youth in the sample had experienced past-year ARBs by year five (*n* = 106; cumulative total). ARB^+^ participants at year five ranged between 18 and 26 years, with the majority (59.5%) reporting more than one past-year ARB and 56.6% reporting ARBs as lasting longer than 1 h in duration. In a series of longitudinal analyses, these data are the first to demonstrate the unique effects of ARBs on cognitive development in adolescence, specifically in domains of executive function and learning and memory. Notably, these effects were apparent even after controlling for frequency of alcohol use. Results showing subtle patterns of ARB-related cognitive change may ultimately inform future efforts to prevent adverse alcohol use consequences among younger drinkers.

### Alcohol-Related Blackouts in the National Consortium on Alcohol and Neurodevelopment in Adolescence Sample

The prevalence rates of ARBs in the NCANDA sample are lower than those reported in other literature, which estimates that among emerging adults and college students, past-year ARB incidence is at least 29.2% for females and 28.8% for males (65). Our finding of lower ARB prevalence likely reflects the NCANDA study design, which limited heavy drinkers at baseline. Nonetheless, the present findings suggest that a small group of ARB^+^ participants (11.60%) repeatedly experience high ARB frequencies over time, consistent with the literature (5, 66, 67). Additionally, while CDDR items used to measure ARB history did not distinguish between ARB type, en bloc (i.e., EB; complete amnesia for a drinking event) versus fragmentary (i.e., FB; partial amnesia for a drinking event), Miller et al. (68) noted that emerging adults define EBs as an inability to remember details of a drinking event for episodes of more than 1 h. Thus, our findings that more than half of ARB^+^ participants reported ARB durations longer than 1 h suggest that EBs may be more prevalent than expected (69).

With regard to demographic factors, findings that ARB^+^ participants were significantly older and more likely to be White have been reported in other literature, particularly among college students (67, 70, 71). The association between older age and ARBs at each timepoint may reflect NCANDA inclusion criteria which allowed a subset of older participants to exceed alcohol use thresholds (31). While older age has been reported as a predictor for higher ARB risk, other studies suggest that younger college students expect to and experience more ARBs (5, 72). Results suggesting that ARB^+^ and ARB^–^ participants do not significantly differ by sex have also been previously reported in the literature (65, 72).

### Alcohol-Related Blackouts as a Predictor of Attenuated Cognitive Function

After accounting for demographic factors, a significant relationship was found between alcohol use frequency and episodic facial memory such that greater number of past-year alcohol use days predicted attenuated growth for abilities on immediate and delayed facial recognition tasks which persisted over time. These results are consistent with and expand upon findings from Sullivan et al. (73). In examining the relationship between drinking history variables and cognitive performance in the NCANDA sample at baseline, the authors note that a greater number of lifetime alcohol use days was significantly associated with lower episodic memory composite scores.

Perhaps our most salient and robust longitudinal findings suggested that, as hypothesized, experiencing at least one past-year ARB predicted significantly attenuated growth in neuropsychological task performance above and beyond past-year alcohol use frequency. Results found that ARB^+^ participants demonstrated attenuated growth in delayed episodic facial memory by the first annual follow-up compared to ARB^–^ participants. The interaction between alcohol use frequency and ARB history further predicted attenuated growth in delayed episodic facial memory at the first and second annual follow-ups. Additionally, experiencing at least one past-year ARB significantly predicted attenuated growth in visual incidental learning and delayed recall by the fourth annual follow-up. No significant associations were found between ARB history and RCFT_c_ trial performance or visuospatial and executive function measures. Taken together, ARB-related cognitive change was found in domains related to learning and memory for visual information.

The finding that ARBs predict attenuated episodic facial memory, or recognition memory, over time suggests that underlying neurocognitive correlates of facial memory are particularly sensitive to heavy alcohol use. Interestingly, negative, dose-dependent effects of alcohol intoxication on episodic memory are well-documented, with alcohol administration studies noting that acute alcohol use is associated explicitly with worse performance on facial recognition tasks (74–76). Thus, in the context of prior literature, our results indicate that changes in facial recognition persist beyond the period of alcohol intoxication. Further, a trend for ARB-related changes in facial recognition to remit over time despite persistent alcohol-specific effects in this domain suggests that heavy alcohol use resulting in ARBs alters the brain’s developmental trajectory. In fact, drinkers may be particularly susceptible to underlying neurological changes associated with facial recognition at earlier developmental stages. These results are consistent with prior literature reporting that heavy, early-onset alcohol use primes the brain for future susceptibility to alcohol with regard to increased neuroinflammation, neurocognitive changes, and a tendency to experience more ARBs during future drinking events (20, 77–79). However, our study is the first to utilize ARBs as an indicator of vulnerability to alcohol use in domain of facial episodic memory over time.

Additionally, we found that ARBs predict attenuated growth in performance on visual incidental learning and delayed recall tasks at later timepoints. Given a strong association between heavy alcohol use and ARBs, our results extend cross-sectional literature reporting deficits in non-verbal recall and visuospatial functioning in alcohol dependent adolescents (80). Our findings also suggest that repeated instances of heavy alcohol use may result in cumulative, neurotoxic effects which impact incidental learning and delayed recall for visual information. Consistent with these results, functional neuroimaging studies demonstrate a relationship between ARBs, binge drinking, and neurochemical and morphological brain changes indicative of alcohol-induced neurotoxicity, particularly in brain regions undergoing significant developmental maturation [e.g., the frontal lobes and hippocampus; (18, 19, 81–84)]. A compelling series of studies examining gender differences in neurocognition in adolescent drinkers further suggest that persistent, heavy alcohol use may prevent the use of compensatory strategies and recruitment of neural networks resulting in overt behavioral impairment (26). Behavioral data from alcohol administration studies in animal models also demonstrate an association between binge drinking and persistent forms of memory dysfunction (85). However, our findings are unique in that we found associations between ARBs and behavioral changes over time while controlling for alcohol use in humans.

No significant associations were found between ARB history and performance on measures of executive function. The absence of overt changes on executive tasks is inconsistent with cross-sectional data from Min et al. (24), indicating that ARBs, specifically those of longer duration, were associated with lower executive function performance. However, our results are consistent with findings from Wetherill et al. (86), who, using a neuroimaging paradigm, found no significant relationship between ARBs and behavioral changes in response inhibition despite underlying abnormalities in frontal activation. Negative findings with regard to a relationship between ARB history and executive function are notable in the context of ARBs predicting attenuated development of incidental visual learning and delayed recall function on the RCFT. Performance on the RCFT is mediated, in part, by the frontal lobes, with literature suggesting that poor organization and problem-solving in participants with chronic AUD accounts for lower RCFT_i_ and RCFT_d_ trial performance (87) As such, persistent ARB-related changes in executive abilities may only become apparent as cognitive load increases, given that intact RCFT performance requires more cognitive resources than the PCET. Further, given its incidental learning component, the RCFT requires organization and efficient learning strategies to consolidate, encode, and retrieve visual information properly. A pattern in which ARBs predict changes in RCFT_i_ and RCFT_d_ trial but not RCFT_c_ trial performance suggests that ARBs may be a marker of inefficient learning related to underlying changes in frontally mediated functions rather than difficulties processing visual information. Prior analyses of executive control in the NCANDA sample by Lannoy et al. (39) have also found that despite intact response inhibition, participants with a history of heavy drinking take longer to become proficient at the task at hand suggesting a relationship between heavy alcohol use and inefficient learning in adolescents and emerging adults.

### Limitations and Future Directions

One limitation of this study is the ability to control for the impact of developmental effects on cognitive performance. While age was included in all models as the basis for individually varying times of observation, the impact of brain development, particularly within the frontal lobes, may have resulted in smaller effect sizes and less robust associations between ARBs and cognitive function over time. Indeed, within the NCANDA sample, prior analyses have found that age contributes significantly to performance on all cognitive measures except for episodic and working memory (73). Given a relatively small sample size of ARB^+^ participants, the current analyses may have lacked the power to detect subtle changes in performance growth on executive tasks in particular relative to significant frontal lobe development. Test-retest effects were also not included in growth models, which may have confounded true declines in cognition related to ARBs and heavy drinking given that several of the cognitive measures used in the current study have been shown to have practice effects (1).

Controlling for alcohol use frequency opposed to quantity is another study limitation given that higher alcohol use quantity is associated with ARB frequency (88). It should also be acknowledged that past-year alcohol use frequency was measured annually using a single item which can be susceptible to recall bias. However, we selected past-year alcohol use frequency as measured by the CDDR given that it was used by previous studies and considered a valid and reliable metric (47–49, 73). Finally, a limitation of this study is that we did not control for other substance use, including marijuana use. By design, the NCANDA study largely excluded individuals reporting a significant substance use history at baseline, including those whose reported alcohol use which exceeded age-appropriate levels. Base rates for all other past-year substance use remained low across all years of data collection (i.e., <10% of total sample at fifth annual follow-up; see Brown et al. (31) for detailed summary of substances evaluated at each visit) except for marijuana use. A closer inspection of the data revealed that 52.6% of participants reported past-year marijuana use by the fifth annual follow-up. Therefore, marijuana use could have biased growth curve model estimates and future studies should explore the relationship between marijuana, ARBs, and cognitive changes over time in adolescent drinkers.

Prior literature has demonstrated that ARB variables such as frequency, duration, and type (i.e., EBs versus FBs) yield important information about predictors and consequences of heavy alcohol use (5, 67, 89). While ARB frequency and duration were analyzed cross-sectionally, longitudinal models were limited to including only the dichotomous (yes vs. no) past-year ARB history variable. As such, future longitudinal studies should consider ARB frequency, duration, and type when examining the relationship between ARBs and persistent cognitive change. The distinction between ARB types is particularly important given that EBs and FBs are likely associated with distinct neurocognitive mechanisms and experienced at different rates among adolescents and emerging adults. Experiencing one type of ARB over the other may place younger drinkers at varying levels of risk for persistent cognitive change or perhaps different types of cognitive change.

In addition, subtests of the WebCNP and the RCFT yield both accuracy and response time scores as measures of cognitive function over time. Within the NCANDA sample, alcohol use has been associated with changes in both accuracy and response time scores, with longitudinal changes in response time thought to reflect how efficiently participants perform tasks within each cognitive domain, or their ability to learn the task over time (73, 90, 91). Given that the current analyses focused on accuracy scores, future studies should consider both changes in accuracy and response time for each task to compare which metric best captures development of cognitive abilities in each domain since tasks may differ in the degree to which they are mediated by processing speed. Finally, future studies should expand the current findings by including functional imaging data in longitudinal models or by longitudinally examining the neurocognitive correlates of ARBs using functional imaging paradigms. Identifying specific brain regions associated with the behavioral changes observed in our study will more precisely identify how patterns of heavy drinking impact brain development in adolescents and emerging adults.

## Conclusion

Taken together, our findings highlight ARBs as a predictor of persistent changes in learning and memory for visual information, with ARB-related attenuations in cognitive development unique from cognitive impairment associated with alcohol use alone and notable in younger drinkers. In the context of the NCANDA sample, these results suggest that brain development and subsequent cognitive abilities are highly vulnerable to ARBs during adolescence, with lasting effects on memory functions observed in emerging adults. Distinct changes in cognitive profiles related to ARBs may thus represent an important target for intervention in high-risk adolescents and emerging adults.

## Data Availability Statement

The original contributions presented in the study are included in the article/supplementary material, further inquiries can be directed to the corresponding author.

## Ethics Statement

All participants underwent informed consent procedures in which adult participants or parents of minor participants provided written informed consent and minor participants provided assent. The Institutional Review Boards of each site approved all NCANDA study protocols.

## Author Contributions

SL contributed to conceptualization, formal analysis, and wrote the original draft of the manuscript. FB contributed to data curation, resources, funding acquisition, conceptualization, methodology, review, and editing of the manuscript. EM-O contributed to conceptualization, methodology, review, and editing of the manuscript. AH contributed to conceptualization, review, and editing the manuscript. RW contributed to methodology, formal analysis, review, and editing the manuscript. SS contributed to investigation and review of the manuscript. DC contributed to data curation, resources, funding acquisition, and review and editing of the manuscript. KN contributed to investigation, project administration, review, and editing of the manuscript. SB contributed to funding acquisition, review, and editing of the manuscript. ST contributed to data curation, resources, funding acquisition, review, and editing of the manuscript. TS contributed to conceptualization, methodology, investigation, review, and editing of the manuscript. All authors contributed to the article and approved the submitted version.

## Conflict of Interest

The authors declare that the research was conducted in the absence of any commercial or financial relationships that could be construed as a potential conflict of interest.

## Publisher’s Note

All claims expressed in this article are solely those of the authors and do not necessarily represent those of their affiliated organizations, or those of the publisher, the editors and the reviewers. Any product that may be evaluated in this article, or claim that may be made by its manufacturer, is not guaranteed or endorsed by the publisher.
